# You'll never dry alone: cross‐kingdom metabolic coupling supports the desiccation resilience in a macroalgal holobiont

**DOI:** 10.1111/nph.71557

**Published:** 2026-09-10

**Authors:** José Flores‐Uribe

**Affiliations:** ^1^ Department of Plant‐Microbe Interactions Max Planck Institute for Plant Breeding Research 50829 Cologne Germany

**Keywords:** desiccation tolerance, drought, glycerol‐3‐phosphate (G3P), holobiont, macroalgae, *Pyropia haitanensis*, syncom

## Abstract

This article is a Commentary on Wang *et al*. (2026), **252**: 2070–2087.

Microbial communities can enhance plant drought tolerance by modulating root physiology and stress‐response pathways, sustaining growth, photosynthetic activity, and water use efficiency under drought conditions (Copeland *et al*., 2025). Terrestrial plants face water stress over days or weeks, whereas aquatic plants such as the red macroalga *Pyropia haitanensis* lose up to 95% of their relative water content within hours with each tidal oscillation, often once or twice per day. In an article published in this issue of *New Phytologist*, Wang *et al*. (2026, pp. 2070–2087) report how the microbiota of *P. haitanensis* contributes to intertidal resilience across a desiccation‐rehydration cycle. Their findings reveal coordinated responses at the holobiont level, highlighting that bacteria acting together, rather than individual strains, are crucial for protection.

*That the SynCom outperformed either strain alone points to synergistic contributions, and paired metabolomic and transcriptomic in vitro profiling of the isolates revealed how their strategies differ.*

The authors tracked *Pyropia haitanensis* relative water content and photosynthetic efficiency across eight time points during a field desiccation‐rehydration cycle. In addition, they profiled the taxonomic composition of the epiphytic microbiota to construct a co‐occurrence network and used metagenomic sequencing to assess the functional potential of the thallus microbial community. As thallus relative water content collapsed from 100% to 6% within 5 h and rebounded within 10 min of rehydration, the epiphytic community also experienced pronounced restructuring. Within the first hour of drying, key taxa and their associations were disrupted, destabilizing the microbial network, which was then partially and progressively rebuilt over the remaining hours of desiccation, suggesting adaptive restructuring toward a more stress‐tolerant state. Among the affected taxa, degraders such as *Aquimarina*, *Tenacibaculum*, and *Erythrobacter*, which depend on persistently hydrated, biofilm‐like niches, disappeared earliest. Rehydration drove recruitment of seawater‐derived taxa in the epiphytic community and rapid network reassembly, although the network's initial complexity was not completely restored. This recolonization relied heavily, but not exclusively, on the surrounding reservoir, suggesting that host‐associated filtering influences which bacteria can colonize rather than reflecting passive acquisition of abundant environmental bacteria. Similar restructuring driven by desiccation has been reported in the brown alga *Sargassum thunbergii*, where tidal dehydration reshaped community diversity and predicted functional genes (Sun *et al*., 2026), with some taxon‐level responses even differing between male and female thalli (Sun *et al*., 2022). However, the mechanisms linking these community changes to host resilience remain poorly understood.

As *P. haitanensis* dried, both intracellular and extracellular glycerol‐3‐phosphate (G3P) increased sharply, peaking under severe desiccation, while the metagenome showed coordinated enrichment of bacterial G3P ABC transporters, as well as antioxidant, osmoprotective, and exopolysaccharide biosynthesis genes. In vascular plants such as sorghum, drought‐stressed roots accumulate G3P and enrich for beneficial bacteria that can promote host growth under water stress (Xu *et al*., 2018). The presence of an analogous metabolic bridge in a red macroalga suggests that metabolite‐mediated support of stress‐associated microbiota may represent a recurring strategy across drought‐ and desiccation‐tolerant systems.

To test whether members of the microbiota contributed to intertidal resilience, the authors isolated 1289 strains from the epiphytic community and selected two representatives of key taxa in the network, *Sulfitobacter* and *Alteromonas*, to assess their role in host desiccation tolerance. Both strains grew faster when supplied with G3P in culture, and thalli pretreated with either isolate or with both as a simple synthetic community (SynCom) showed reduced loss of photosynthetic efficiency during drying and faster rehydration than untreated controls. Using heat‐killed bacteria or substituting an unrelated marine strain abolished the benefit, excluding a nonspecific host response to bacterial presence or surface occupation alone. Moreover, reinoculating antibiotic‐treated thalli with either isolate, and particularly with their combination, resulted in increased rehydration compared to the axenic control. That the SynCom outperformed either strain alone points to synergistic contributions, and paired metabolomic and transcriptomic *in vitro* profiling of the isolates revealed how their strategies differ.

*Sulfitobacter* can use host‐derived G3P as a carbon source and channel it into building a protective barrier, supporting osmoprotectant uptake and exopolysaccharide matrix synthesis, while also scavenging reactive oxygen species through catalase activity and accumulation of the polyamine spermidine. *Alteromonas*, by contrast, appears to pursue a metabolic buffering strategy, redirecting resources away from siderophore production and toward arginine biosynthesis, which provides precursors for membrane‐stabilizing polyamines. Metabolomic analyses show that both isolates accumulate jasmonic acid, with *Sulfitobacter* additionally accumulating indole‐3‐acetic acid, raising the possibility that these bacterial compounds contribute to host recovery, analogous to hormone‐associated mechanisms in terrestrial drought‐resistant symbioses. A notable difference is that, while rhizosphere bacteria can induce host‐derived signaling responses (Copeland *et al*., 2025), the algal isolates synthesize these compounds themselves. It would also be interesting to test whether the beneficial effects of these macroalgal isolates extend to land plants, analogous to the complementation observed between the microbiota of *Chlamydomonas reinhardtii* and *Arabidopsis thaliana* (Durán *et al*., 2022). Taken together, these observations point to a holobiont strategy for surviving desiccation in which dehydration reshapes the community toward tolerant taxa such as *Sulfitobacter* and *Alteromonas*, while G3P secreted by *P. haitanensis* may support these bacteria and the functions that stabilize the partnership, suggesting a cross‐domain metabolic coupling that promotes resilience to intertidal stress.

Going forward, the bacterial culture collection established by Wang *et al*. opens new opportunities to move from observing macroalgal microbiota to experimentally testing their functions. A logical next step would be to assemble increasingly complex SynComs and ask how community composition shapes stress protection, functional redundancy, and microbe–microbe interactions. Genome sequencing of the collection would enable the rational design of these SynComs based on metabolic modelling predictions (Mataigne *et al*., 2022), while targeted mutants of key taxa could pinpoint the bacterial determinants of the stress‐attenuating effects. These bottom‐up approaches have proven to be a powerful strategy for advancing plant–microbiota research, for example, by identifying principles of community assembly (Vorholt *et al*., 2017; Carlström *et al*., 2019), and extending them to macroalgal systems could similarly deepen our understanding of these holobionts.

By revealing how microbial partners contribute to the desiccation resilience of *P. haitanensis*, Wang *et al*. show how host–microbe interactions can shape stress responses in macroalgal holobionts.

## Disclaimer

The New Phytologist Foundation remains neutral with regard to jurisdictional claims in maps and in any institutional affiliations.
